# The association of body composition with abdominal aortic aneurysm growth after endovascular aneurysm repair

**DOI:** 10.1186/s13244-022-01187-7

**Published:** 2022-04-25

**Authors:** Ge Hu, Ning Ding, Zhiwei Wang, Zhengyu Jin

**Affiliations:** 1 Department of Radiology, State Key Laboratory of Complex Severe and Rare Disease, Peking Union Medical College Hospital, Chinese Academy of Medical Sciences and Peking Union Medical College, Beijing, 100730 China; 2 Medical Research Center, Peking Union Medical College Hospital, Chinese Academy of Medical Sciences and Peking Union Medical College, Beijing, 100730 China

**Keywords:** Abdominal aortic aneurysm, Endovascular aneurysm repair, Body composition, Muscle, Adipose tissue

## Abstract

**Background:**

Body composition (BC) may be associated with abdominal aortic aneurysm (AAA) growth, but the results of previous research are contradictory. This study aimed to explore the relationship between BC and postoperative aneurysm progression.

**Methods:**

Patients with regular postoperative follow-ups were retrospectively identified. The volume change of the aneurysm was measured to evaluate AAA progression. After segmenting different body components (subcutaneous fat, visceral fat, pure muscle, and intramuscular fat), the shape features and gray features of these tissues were extracted. Uni- and multivariable methods were used to analyze the relationship between imaging features of BC and AAA growth.

**Results:**

A total of 94 patients (68 ± 8 years) were eligible for feature analyses. Patients with expansive aneurysms (29/94; volume change > 2%) were classified into Group(+) and others with stable or shrunken aneurysms (65/94) were classified into Group(−). Compared with Group(+), Group(−) showed a higher volume percent of pure muscle (21.85% vs 19.51%; *p* = .042) and a lower value of intramuscular fat (1.23% vs 1.65%; *p* = .025). CT attenuation of muscle tissues of Group(−) got a higher mean value (31.16 HU vs 23.92 HU; *p* = .019) and a lower standard deviation (36.12 vs 38.82; *p* = .006) than Group(+). For adipose tissue, we found no evidence of a difference between the two groups. The logistic regression model containing muscle imaging features showed better discriminative accuracy than traditional factors (84% vs 73%).

**Conclusions:**

Muscle imaging features are associated with the volume change of postoperative aneurysms and can make an early prediction. Adipose tissue is not specifically related to AAA growth.

**Supplementary Information:**

The online version contains supplementary material available at 10.1186/s13244-022-01187-7.

## Key points

- Patients with shrunken aneurysms had more muscle and less intramuscular fat.
- Muscles of patients with shrunken aneurysms got a higher CT value.
- Adipose tissue is not specifically related to abdominal aortic aneurysm growth.
- Muscle imaging features can make a better prediction for aneurysm progression.

## Background

Abdominal aortic aneurysm (AAA) is a common cardiovascular disease with high mortality [1]. About 150,000–200,000 deaths in the world are associated with AAA every year [2]. Guidelines recommend a surgical operation for the aneurysm with a maximum diameter larger than 5.5 cm (for men) or 4.5 cm (for women), and endovascular aneurysm repair (EVAR) is usually a suitable choice for AAA [3, 4]. Although EVAR is safer and minimally invasive compared to traditional open repair, there is still a potential risk of enlargement even rupture of aneurysm after intervention [5]. Therefore, exploring the factors related to the progression of postoperative AAA is important to the surveillance and treatment of patients with AAA.

Body composition (BC) such as fat and muscle are associated with the development of various cardiovascular diseases [6, 7]. A higher body mass index (BMI) and particularly fat mass index are related to increased risk of aortic valve stenosis, heart failure, and most other cardiovascular conditions [6]. Low muscle mass or strength is a risk factor of major cardiovascular events and is associated with increased cardiovascular disease mortality in individuals aged ≥ 65 years [7]. However, reviewing research on the relationship between BC and AAA, the results are contradictory [8–10]. Several studies proved that obesity was positively associated with AAA presence or growth [10], but others came to conclusions opposed to the former and demonstrated no association between them [11, 12]. Moreover, some research found a significant link between low skeletal muscle mass and mortality in patients after repair [13, 14], but others provided this association could not be replicated [15, 16].

These contradicting results may be caused by the following reasons: (a) Differences in objects and indicators. Some studies evaluated overall obesity through BMI or abdominal circumference, and others assessed center obesity through subcutaneous fat or visceral fat [9–12]. (b) Most research of muscles focused on the relationship between the psoas muscle and AAA progression but ignored the influence of anterior and lateral muscle groups of the abdomen [8]. (c) Previous studies based on CT scans mostly extracted features from a certain axial slice rather than continuous multilayer images [8, 11, 12]. Furthermore, few studies involve discussion of CT value. However, gray features, such as the mean value of CT attenuation which describes the distribution of intensities within the image region, may also influence the aneurysm progression as same as shape features.

In this study, we selected consecutive multilayer slices from the first postoperative follow-up CT scans and segmented various body components (subcutaneous fat, visceral fat, pure muscle, and intramuscular fat). Through analyzing multiple shape features and gray features of these tissues and compared with conventional factors (endoleak), we explored the relationship between BC and the volume change of aneurysms to realize the early prediction of AAA growth after EVAR.

## Methods

This study was approved by the institutional review board and conducted following the Declaration of Helsinki. The requirement for informed consent was waived by the institutional review board given the retrospective nature of the study.

### Study patients

**Fig. 1:**
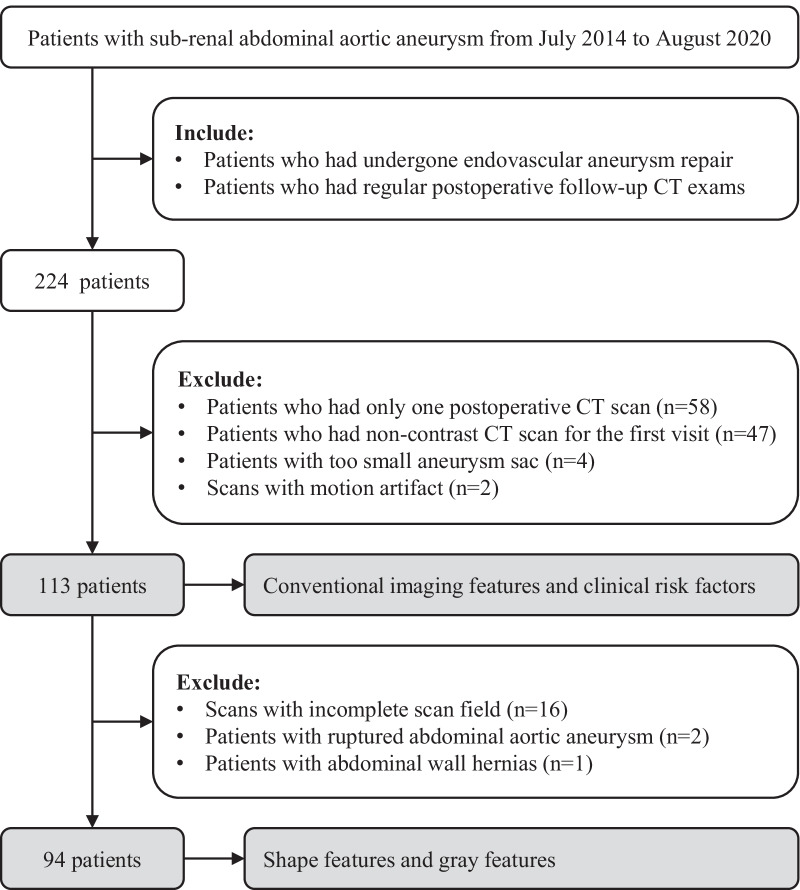
Patient selection flowchart

We collected patients with sub-renal AAA from July 2014 to August 2020 by querying the electronic medical records system. Inclusion criteria were: (a) patients who had undergone EVAR; (b) at least two regular postoperative follow-up CT examinations (approximately 3 and 12 months). Exclusion criteria were: (a) only one postoperative CT scan (*n* = 58); (b) non-contrast CT examinations for the first follow-up visit (*n* = 47); (c) too small aneurysm sac (*n* = 4); and (d) scans with motion artifacts (*n* = 2). According to the above criteria, a total of 113 patients were identified (Fig. 1). We performed traditional imaging evaluation on their CT images and gathered clinical materials (past disease histories, living habits, and metabolism-related indexes) recorded before surgery (Table 1).

**Table 1:** Conventional imaging features and clinical risk factors

Characteristics	All	Group(+)	Group(−)	*p* value
*Demographic factors*				
Sex	*M* = 101; *W* = 12	*M* = 31; *W* = 5	*M* = 70; *W* = 7	.657
Age (years)	68 ± 8	70 ± 8	67 ± 9	.150
*Conventional imaging features*				
Volume change (%)	− 2.37 ± 13.40	10.70 ± 9.96	− 8.49 ± 10.01	–
*V*_1_ (cm^3^)	114.60 (83.90, 177.55)	108.20 (80.50, 171.98)	117.00 (83.90, 180.85)	.739
*V*_2_ (cm^3^)	108.10 (77.45, 164.30)	119.60 (90.70, 183.65)	106.00 (73.05, 162.45)	.208
Maximal diameter (mm)	49.00 (39.50, 57.15)	49.70 (40.13, 61.08)	48.00 (39.35, 54.70)	.318
CT-reported endoleak	Yes = 19; No = 94	Yes = 12; No = 24	Yes = 7; No = 70	.001*
*Past disease histories*				
Hypertension	Yes = 71; No = 39; NR = 3	Yes = 23; No = 11; NR = 2	Yes = 48; No = 28; NR = 1	.649
Hypertension duration (years)	5 (0, 15)	10 (0, 20)	4 (0, 10)	.123
SP (mmHg)	138 (128, 158)	143 (129, 163)	138 (128, 156)	.432
DP (mmHg)	80 (72, 90)	80 (69, 90)	80 (73, 90)	.488
Heart disease	Yes = 34; No = 79	Yes = 15; No = 21	Yes = 19; No = 58	.067
Diabetes	Yes = 16; No = 96; NR = 1	Yes = 7; No = 29	Yes = 9; No = 67; NR = 1	.283
*Living habits*				
Smoking history	Yes = 71; No = 41; NR = 1	Yes = 24; No = 12	Yes = 47; No = 29; NR = 1	.621
Current smoking status	Yes = 39; No = 72; NR = 2	Yes = 14; No = 21; NR = 1	Yes = 25; No = 51; NR = 1	.466
Smoking duration (years)	30 (0, 40)	30 (0, 40)	20 (0, 40)	.671
Alcohol consumption	Yes = 38; No = 73; NR = 2	Yes = 17; No = 18; NR = 1	Yes = 21; No = 55; NR = 1	.031*
Current alcohol consumption status	Yes = 25; No = 86; NR = 2	Yes = 10; No = 25; NR = 1	Yes = 15; No = 61; NR = 1	.301
*Metabolism-related*				
Total cholesterol (mmol/L)	4.04 (3.52, 4.87)	4.11 (3.45, 5.13)	4.03 (3.53, 4.79)	.851
Triglyceride (mmol/L)	1.31 (0.95, 2.00)	1.29 (0.96, 2.22)	1.35 (0.95, 1.90)	.696
HDLC (mmol/L)	0.91 (0.76, 1.04)	0.93 (0.81, 1.22)	0.90 (0.74, 1.03)	.183
LDLC (mmol/L)	2.40 (1.94, 2.97)	2.49 (1.70, 2.99)	2.37 (1.98, 2.96)	.880

Continuous variables are presented as mean ± standard deviation or median (interquartile range). Categorical variables are presented as numbers. Group(+) represents patients with an aneurysm expansion, and Group(−) represents patients with a stable or shrunken aneurysm

*V*_1_ first postoperative follow-up aneurysm volume, *V*_2_ second follow-up aneurysm volume, *SP* systolic pressure, *DP* diastolic pressure, *HDLC* high-density lipoprotein cholesterol, *LDLC* low-density lipoprotein cholesterol, *NR* not reported

*p values in bold highlight the figures less than .05

To obtain accurate segmentation results of abdominal BC, we further screened the first follow-up scans of the enrolled patients. Exclusive criteria were: (a) incomplete scan field (*n* = 16); (b) ruptured AAA (*n* = 2); and (c) abdominal wall hernias (*n* = 1). Finally, 94 patients were eligible for analysis of shape features and gray features (Fig. 1).

### Conventional imaging evaluation

**Fig. 2:**
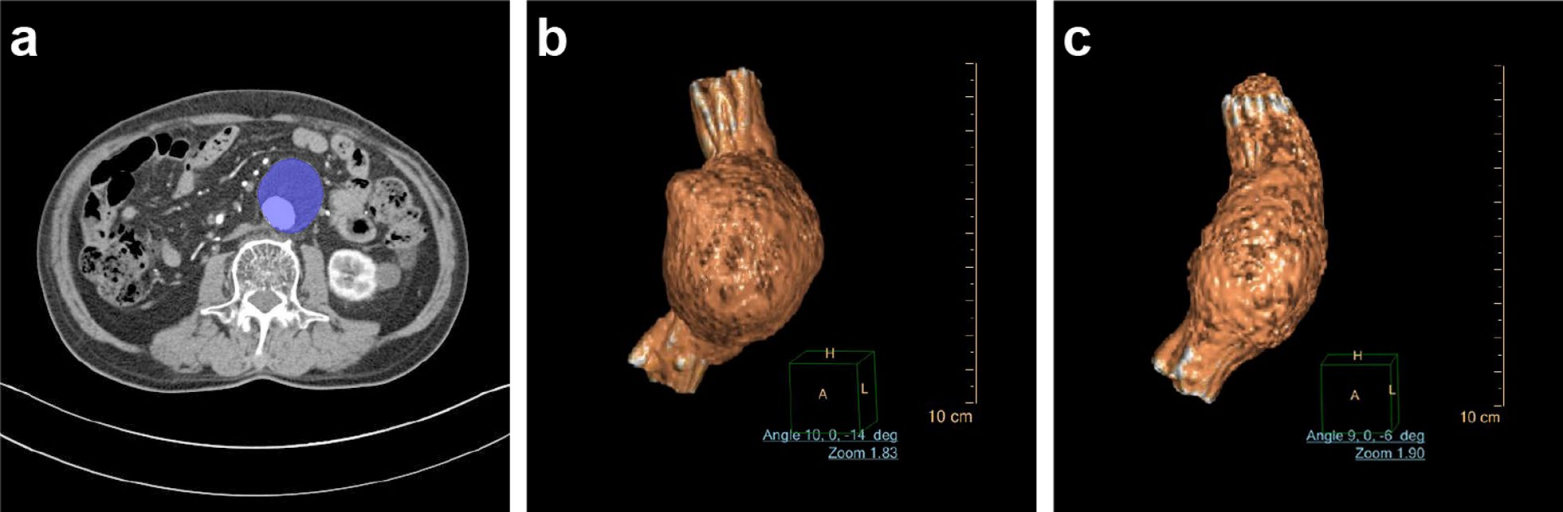
Traditional imaging evaluation. **a** Maximal axial plane of the abdominal aortic aneurysm (the blue area). **b**, **c** Reconstruction and volume measurement of the aneurysm based on the first and the second postoperative follow-up CT scans

Conventional imaging features consisted of the first postoperative follow-up aneurysm volume (*V*_1_), the second follow-up aneurysm volume (*V*_2_), the first follow-up maximum aneurysm diameter, and the presence or absence of endoleaks. The aneurysm volume was measured from the level of the lower renal artery to the level of bifurcation on both sides [17, 18]. Two radiologists with 10 and 6 years of experience performed the measurement (Fig. 2). Details for evaluation procedure and CT protocols are described in Additional file 1.

The evaluation standard of AAA growth was derived from the volume change between *V*_1_ and *V*_2_ [19, 20]. If (*V*_2_ − *V*_1_)/*V*_1_ was greater than 2%, it was considered an expansive aneurysm. Otherwise, it was a stable or shrunken aneurysm.

### Body composition segmentation

**Fig. 3:**
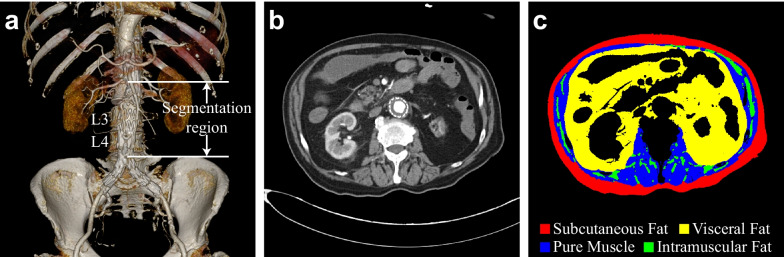
Body composition segmentation. **a** The region of segmentation images (from the lower renal artery level to the upper edge of the iliac bone). **b** An original CT slice. **c** Body composition segmentation results of image **b**

Abdominal BC was segmented from the first postoperative follow-up CT slices (Fig. 3). The segmentation range was from the lower renal artery level to the upper edge of the iliac bone. The segmentation targets were subcutaneous fat, visceral fat, pure muscle, and intramuscular fat. Moreover, we also obtained the area of total adipose tissue (subcutaneous fat + visceral fat) and total muscle tissue (pure muscle + intramuscular fat).

In this study, we used a semiautomatic segmentation procedure. Firstly, the threshold method was adopted to separate fat and muscle from the abdominal images. The CT attenuation of adipose tissue was defined from − 190 to – 30 HU, and the muscle was defined from − 30 to 150 HU [21, 22]. Meanwhile, the morphological operation was used to eliminate noise and void. After preliminary segmentation, subcutaneous fat was further segmented from adipose tissue by using a level set method [23]. Considering the complexity and diversity of abdominal images, we corrected segmentation results manually to remove the interference structures which cannot be segmented correctly only depending on the automatic process.

### Shape features and gray features

Imaging features analyzed in this study were comprised of shape features and gray features. Shape features contained the volume and average area of each body component, and the average abdominal circumference. Gray features contained mean value, standard deviation, kurtosis, and skewness of CT value of BC voxels (Additional file 1).

To eliminate the bias caused by individual variations, the volume percent was finally analyzed instead of the original volume data. Volume percent (%) = original volume (cm^3^)**/**total volume of abdomen (cm^3^) × 100%. We also computed the volume ratio between different body components. Average area and circumference were both adjusted for height [24]. Adjusted average area (cm^2^/m^2^) = unadjusted area (cm^2^)**/**height^2^ (m^2^). Adjusted average circumference (cm/m) = unadjusted circumference (cm)**/**height (m). The process of image segmentation and feature extraction was realized by MATLAB (version R2020b; Mathworks, Natick, Massachusetts) programming.

### Statistical analysis

Clinical materials, traditional imaging characteristics, shape features, and gray features were analyzed in this study. In univariable analysis, the Shapiro–Wilk test was used to assess the normality of distribution. Continuous variables were analyzed using the Student’s *t* test or Mann–Whitney *U* test, and categorical variables were compared using the Chi-square test or Fisher exact test.

Variables with *p* values less than 0.10 were used in subsequent logistic regression analysis. The variable selection method was stepwise regression based on maximum likelihood estimation. Omnibus test and Hosmer–Lemeshow test were used to evaluate the logistic regression model (LRM). Odds ratio (OR) with 95% CI of variables was recorded. The prediction probability of LRM was further assessed using the AUC value.

*p* values more than 0.05 represented the high goodness of fit of a model in the Hosmer–Lemeshow test. In other statistical tests, *p* values less than 0.05 were considered to indicate significant differences. Data analysis was completed using SPSS (version 26.0; IBM, Armonk, NY). Statistical power was evaluated by PASS (version 15.0; NCSS, Kaysville, Utah).

## Results

Inter-observer reproducibility between the two radiologists was excellent for measurements of *V*_1_ (ICC = 0.986), *V*_2_ (ICC = 0.985), maximal diameter (ICC = 0.956), and endoleak (ICC = 0.932). The results from one of the two radiologists were used for further analysis.

### Patient characteristics

Table 1 provides conventional imaging features and clinical risk factors of 113 patients (68 ± 8 years; 101 men, 12 women). These patients were divided into two groups according to the AAA growth. Patients with expansive aneurysms were classified into Group(+) and others with stable or shrunken aneurysms were classified into Group(−).

Group(+) contained 36 patients with an average volume change of 10.7% and a maximal diameter of 49.7 mm. Group(−) contained 77 patients with an average volume change of − 8.5% and a maximal diameter of 48.0 mm. 12 endoleaks (type II) in Group(+) and 7 endoleaks (type II) in Group(−) were reported in the first postoperative CT scans. As shown in Table 1, CT-reported endoleak (33% [12/36] vs 9% [7/77]; *p* = 0.001) and alcohol consumption (49% [17/35] vs 28% [21/76]; *p* = 0.03) showed significant differences between the two groups.

### Shape feature analysis

Analysis results of shape features are presented in Table 2. Among the 94 patients (the selection flowchart is shown in Fig. 1), 29 of them (31%) were classified into Group(+) and 65 of them (69%) were classified into Group(−). Compared with Group(+), Group(−) showed a higher volume percent of pure muscle (21.85% vs 19.51%; *p* = 0.042) and a lower value of intramuscular fat (1.23% vs 1.65%; *p* = 0.025). The volume ratio of intramuscular fat**/**pure muscle of Group(−) was also significantly lower than Group(+) (0.06 vs 0.08; *p* = 0.011). Statistics of the average area reflected the same tendency. Group(−) has a larger area of pure muscle (46.71 cm^2^/m^2^ vs 45.52 cm^2^/m^2^; *p* = 0.465) and a smaller area of intramuscular fat (2.62 cm^2^/m^2^ vs 3.96 cm^2^/m^2^; *p* = 0.014).

**Table 2:** Shape feature analysis of abdominal body composition

Shape features	All	Group(+)	Group(−)	*p* value
*Volume percent (%)*				
Subcutaneous fat	19.52 ± 5.28	20.24 ± 4.92	19.21 ± 5.45	.384
Visceral fat	25.77 (21.11, 29.71)	24.90 (20.60, 29.95)	25.85 (21.01, 29.61)	.918
Pure muscle	21.39 (18.69, 23.38)	19.51 (18.05, 22.70)	21.85 (19.31, 23.63)	.042*
Intramuscular fat	1.39 (0.95, 2.02)	1.65 (1.03, 2.58)	1.23 (0.88, 1.94)	.025*
Total fat	46.62 (41.06, 50.87)	47.82 (40.10, 54.11)	46.35 (42.50, 50.70)	.661
Total muscle	22.62 (20.31, 24.94)	21.69 (19.77, 24.08)	23.76 (20.77, 24.98)	.111
*Volume ratio*				
Subcutaneous fat**/**Visceral fat	0.78 (0.61, 1.03)	0.83 (0.60, 1.03)	0.78 (0.61, 1.00)	.569
Intramuscular fat**/**Pure muscle	0.07 (0.04, 0.10)	0.08 (0.05, 0.18)	0.06 (0.04, 0.09)	.011*
Total fat**/**Total muscle	2.02 (1.72, 2.49)	2.24 (1.64, 2.66)	2.01 (1.74, 2.40)	.406
*Average area* (cm^2^/m^2^)				
Subcutaneous fat	44.23 ± 15.45	46.68 ± 12.69	43.26 ± 16.41	.351
Visceral fat	57.40 ± 23.99	59.48 ± 25.42	56.58 ± 23.56	.612
Pure muscle	46.37 ± 6.83	45.52 ± 6.66	46.71 ± 6.91	.465
Intramuscular fat	2.88 (2.13, 4.40)	3.96 (2.54, 5.74)	2.62 (2.12, 4.00)	.014*
Total fat	107.77 (81.51, 125.27)	116.73 (80.48, 124.94)	104.53 (82.90, 125.63)	.608
Total muscle	49.83 ± 7.02	49.79 ± 6.99	49.84 ± 7.09	.974
Abdomen	220.96 ± 39.69	226.61 ± 37.52	218.72 ± 40.58	.403
*Circumference* (cm/m)				
Abdomen	57.58 (53.96, 60.72)	59.14 (54.64, 60.60)	56.95 (53.44, 60.94)	.526

Unless otherwise specified, data are mean ± standard deviation or median (interquartile range). Group(+) represents patients with an aneurysm expansion, and Group(−) represents patients with a stable or shrunken aneurysm

*p values in bold highlight the figures less than .05

For adipose tissue, we found no evidence of a difference between the two groups. Subcutaneous fat of Group(+) was numerically higher than Group(−) in both volume percent (20.24% vs 19.21%; *p* = 0.384) and average area (46.68 cm^2^/m^2^ vs 43.26 cm^2^/m^2^; *p* = 351). However, visceral fat of Group(+) showed a higher average area (59.48 cm^2^/m^2^ vs 56.58 cm^2^/m^2^; *p* = 0.612) but a lower volume percent (24.90% vs 25.85%; *p* = 0.918) than Group(−), which showed a conflicting result.

Furthermore, Group(+) showed a larger value of total fat–muscle ratio (2.24 vs 2.01; *p* = 0.406) and a greater degree of abdominal obesity (abdominal area, 226.61 cm^2^/m^2^ vs 218.72 cm^2^/m^2^, *p* = 0.403; abdominal circumference, 59.14 cm/m vs 56.95 cm/m, *p* = 0.526), which was consistent with previous studies. Unadjusted data of shape features are shown in Additional file 1: Tables S1, S2.

### Gray feature analysis

Table 3 provides the results of gray features. Notably, all the data of pure muscle and total muscle tissue showed statistical significance. The total muscle tissue of Group(−) got a higher mean value (31.16 HU vs 23.92 HU; *p* = 0.019) and a lower standard deviation (36.12 vs 38.82; *p* = 0.006). Similarly, pure muscle of Group(−) also showed a higher mean value (36.90 HU vs 34.37 HU; *p* = 0.037) and a lower standard deviation (26.65 vs 27.68; *p* = 0.005). These results illustrated that the muscle tissue of patients with stable or shrunken aneurysms tended to get a greater CT value and smaller dispersion than those with expansive aneurysms.

**Table 3:** Gray feature analysis of abdominal body composition

Gray features	All	Group(+)	Group(−)	*p* value
*Mean value* (HU)				
Subcutaneous fat	− 103.83 (− 107.89, − 97.34)	− 102.94 (− 106.76, − 97.99)	− 104.20 (− 108.16, − 96.68)	.964
Visceral fat	− 96.40 (− 99.55, − 91.75)	− 95.20 (− 99.70, − 92.71)	− 96.56 (− 99.62, − 91.33)	.655
Pure muscle	36.12 ± 5.44	34.37 ± 5.24	36.90 ± 5.39	.037*
Intramuscular fat	− 66.29 ± 4.09	− 67.29 ± 4.28	− 65.85 ± 3.95	.114
Total fat	− 99.27 (− 103.05, − 94.67)	− 99.01 (− 103.18, − 96.75)	− 99.86 (− 103.09, − 93.17)	.854
Total muscle	30.20 (23.35, 34.04)	23.92 (20.75, 32.66)	31.16 (26.16, 36.13)	.019*
*Standard deviation*				
Subcutaneous fat	22.24 (20.84, 23.56)	22.94 (20.47, 24.32)	22.03 (20.86, 23.40)	.835
Visceral fat	24.50 ± 2.01	24.70 ± 2.17	24.42 ± 1.95	.537
Pure muscle	27.00 (26.15, 28.23)	27.68 (27.01, 28.46)	26.65 (25.97, 28.08)	.005*
Intramuscular fat	23.25 ± 2.18	23.66 ± 2.31	23.07 ± 2.12	.225
Total fat	23.93 ± 2.08	23.99 ± 2.20	23.91 ± 2.04	.872
Total muscle	36.96 ± 4.46	38.82 ± 4.66	36.12 ± 4.14	.006*
*Kurtosis*				
Subcutaneous fat	4.19 (3.77, 4.69)	4.15 (3.80, 4.70)	4.23 (3.61, 4.67)	.990
Visceral fat	3.04 (2.83, 3.35)	2.99 (2.76, 3.58)	3.08 (2.87, 3.34)	.542
Pure muscle	3.75 ± 0.37	3.65 ± 0.28	3.79 ± 0.39	.048*
Intramuscular fat	2.68 (2.45, 2.94)	2.58 (2.38, 2.77)	2.75 (2.48, 2.98)	.040*
Total fat	3.39 ± 0.45	3.41 ± 0.48	3.38 ± 0.44	.736
Total muscle	5.16 ± 0.83	4.79 ± 0.80	5.32 ± 0.80	.004*
*Skewness*				
Subcutaneous fat	1.12 (0.92, 1.24)	1.12 (0.98, 1.23)	1.13 (0.87, 1.24)	.847
Visceral fat	0.58 (0.38, 0.74)	0.56 (0.39, 0.74)	0.59 (0.37, 0.74)	.964
Pure muscle	− 0.19 ± 0.29	− 0.09 ± 0.24	− 0.23 ± 0.30	.023*
Intramuscular fat	− 0.55 (− 0.65, − 0.45)	− 0.50 (− 0.60, − 0.43)	− 0.56 (− 0.70, − 0.48)	.054
Total fat	0.82 (0.64, 0.90)	0.80 (0.67, 0.88)	0.84 (0.62, 0.90)	.945
Total muscle	− 1.14 (− 1.23, − 0.95)	− 1.04 (− 1.19, − 0.87)	− 1.15 (− 1.31, − 1.00)	.040*

Unless otherwise specified, data are mean ± standard deviation or median (interquartile range). Group(+) represents patients with an aneurysm expansion, and Group(−) represents patients with a stable or shrunken aneurysm

*HU* Hounsfield unit

*p values in bold highlight the figures less than .05

Moreover, compared with Group(+), the muscle tissue of Group(−) had a higher kurtosis (total muscle, 5.32 vs 4.79, *p* = 0.004; pure muscle, 3.79 vs 3.65, *p* = 0.048) and a greater absolute value of skewness (total muscle, − 1.15 vs − 1.04, *p* = 0.040; pure muscle, − 0.23 vs − 0.09, *p* = 0.023), which further proved the conclusion from the gray features of mean value and standard deviation.

**Fig. 4:**
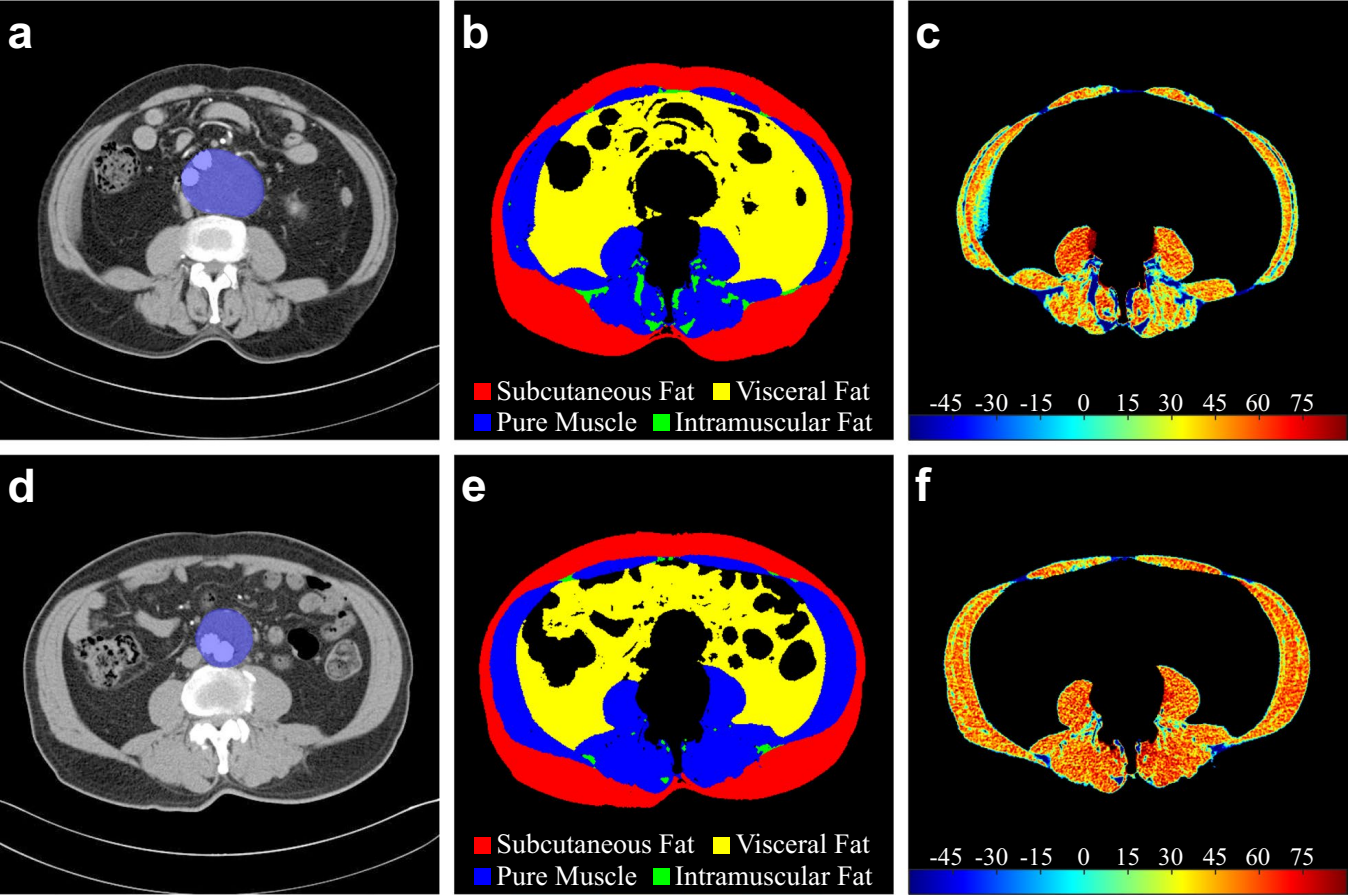
Examples of abdominal aortic aneurysm growth. **a–c** A patient with aneurysm expansion. **d–f** A patient with a shrunken aneurysm. **a** Maximal axial plane of the aneurysm (the blue area). **b** Body composition segmentation results of image a. **c** Pseudocolor image of the total muscle region (intramuscular fat and pure muscle). The red area reflects the high CT value, and the blue reflects the low value (Hounsfield Unit). **d**–**f** represent the same meaning as **a**–**c**

Figure 4 gives two examples of different AAA growth after EVAR. Figure 4a–c shows characteristics of a patient with an expansion AAA in Group(+), and Fig. 4d–f shows a patient with a shrunken AAA in Group(−).

### Logistic regression analysis

Features with *p* values less than 0.10 were used in multivariable analysis. We evaluated three LRMs for comparison. LRM1 included CT-reported endoleak and clinical factors. LRM2 included shape features and gray features. LRM3 contained all factors of the first two models.

As shown in Table 4, endoleak (OR = 5.10, *p* = 0.003) and drinking history (OR = 2.43, *p* = 0.047) retained statistical differences in LRM1. The volume ratio of intramuscular fat**/**pure muscle (OR = 8.89, *p* = 0.014), the mean value of total muscle (OR = 0.04, *p* = 0.029), standard deviation (OR = 7.62, *p* = 0.002), and kurtosis (OR = 0.07, *p* = 0.035) of pure muscle retained statistical differences in LRM2. Endoleak (OR = 5.17, *p* = 0.036), intramuscular fat**/**pure muscle (OR = 9.83, *p* = 0.014), standard deviation (OR = 6.36, *p* = 0.005), and kurtosis (OR = 0.08, *p* = 0.045) of pure muscle were eventually selected in LRM3. The OR values indicated that patients with more intramuscular fat or less muscle tissue had a high risk of aneurysm dilation.

**Table 4:** Multivariable analysis based on the logistic regression model

Multivariable analysis	LRM 1		LRM 2		LRM 3	
	OR (95% CI)	*p* value	OR (95% CI)	*p* value	OR (95% CI)	*p* value
CT-reported endoleak	5.10 (1.74, 14.74)	.003*	–	–	5.17 (1.12, 23.89)	.036*
Alcohol consumption	2.43 (1.01, 5.82)	.047*	–	–	NR	NR
Volume ratio of intramuscular fat**/**pure muscle	–	–	8.89 (1.57, 50.48)	.014*	9.83 (1.60, 60.47)	.014*
Mean value of total muscle	–	–	0.04 (0.00, 0.73)	.029*	0.06 (0.00, 1.05)	.054
Standard deviation of pure muscle	–	–	7.62 (2.17, 26.70)	.002*	6.36 (1.73, 23.36)	.005*
Kurtosis of pure muscle	–	–	0.07 (0.01, 0.83)	.035*	0.08 (0.01, 0.94)	.045*

*LRM* logistic regression model, *OR* odds ratio, *CI* confidence interval, *NR* not reported

*p values in bold highlight the figures less than .05

Table 5 shows the evaluation index of the three LRMs. All of the three models were statistically significant (Omnibus test, *p* < 0.001) with a high goodness of fit (Hosmer–Lemeshow test, *p* > 0.05). LRM2 showed a better discriminative performance than LRM1 (accuracy, 81% vs 73%; AUC, 0.79 vs 0.67), and LRM3 got the best classification results (accuracy = 84%; AUC = 0.81).

**Table 5:** Evaluation of logistic regression model for abdominal aortic aneurysm growth

Evaluation index	LRM 1	LRM 2	LRM 3
The *p* value of Omnibus test	< .001*	< .001*	< .001*
The *p* value of Hosmer and Lemeshow test	> .999	.903	.935
Percentage accuracy in classification (%)	73%	81%	84%
AUC (95% CI)	0.67 (0.55, 0.80)	0.79 (0.69, 0.90)	0.81 (0.70, 0.91)
The *p* value of AUC	.009*	< .001*	< .001*
Sensitivity	64%	71%	75%
Specificity	63%	78%	73%
Cutoff value	0.27	0.50	0.48

*LRM* logistic regression model, *AUC* area under the curve, *CI* confidence interval

*p values less than .05

## Discussion

In this study, we analyzed various imaging features of BC extracted from early follow-up CT scans to explore the association between BC and AAA growth after EVAR. Statistics of shape features showed that patients with stable or shrunken aneurysms had a significantly higher proportion of pure muscle and less intramuscular fat. Previous studies proposed that the muscle mass was an effective parameter reflecting frailty or sarcopenia, which was associated with postoperative recovery [25]. Thus, maintaining muscle strength and quality through exercise, such as resistance training, may benefit patients with AAA after repair.

Results of gray features showed that the CT attenuation (mean value, kurtosis, and skewness) of muscle tissues of Group(−) was higher than Group(+), which illustrated that the muscles of patients with stable or shrunken aneurysms presented a greater degree of leptokurtic (higher peak value) and negative-skewness (more data is greater than the mean value) distribution. Due to the muscle enhancement caused by the contrast medium being greater than adipose tissue, it was reasonable that patients with more muscle tissue got greater CT attenuation, which also showed a good agreement between the results of shape features and gray features.

Another important significance of the gray feature is that it may provide a more convenient and accurate risk stratification for clinics. The volume change is currently the most recognized indicator for aneurysm rupture [19], but traditional imaging measurement is time-consuming with the high requirement for segmentation accuracy. Gray features can be acquired easily in a convenient way, such as sampling from a partial region of the target tissue. The radiologist can select a square or circular region from the abdominal muscle groups of the CT slices, and then, the gray features of these partial images can be automatically extracted for further analysis.

We analyzed three LRMs that contained different variables. LRM2 containing shape and gray features got a relatively accurate classification compared with LRM1 (81% vs 73%). The results illustrated that the muscle imaging features not only relate to the aneurysm volume change but also can provide an early prediction for AAA growth. LRM3 containing all factors showed the best accuracy (84%) of the three models, which meant the richer the features involved in the model, the higher the prediction accuracy.

Be noted that we found no evidence of differences among the imaging features of adipose tissue between the two groups. Given the incompatible conclusions of previous research, insignificant results in our study are entirely predictable. In addition, the visceral fat of Group(+) in the statistical results of shape features showed a higher average area but a lower volume percent. This conflicting result was similar to the studies mentioned in the Introduction. Different indicators produced different conclusions, which further proved that adipose tissue may be irrelevant to the aneurysm growth. Currently, many scholars analyzed the fat around aneurysms from other aspects, such as the inflammatory gene expression [26] and autoimmune response signatures [27], and found many meaningful results. Perhaps these factors can better reveal the influence of fat on aneurysm growth.

The presence of endoleaks is usually considered a major driver of AAA expansion after surgery. In this paper, a total of 19 endoleaks (type II) were reported in the first follow-up CT scans. We compared the shape and gray features of different body components between the patients with and without CT-reported endoleaks. There were no significant differences in results of subcutaneous fat (*p* = 0.261), visceral fat (*p* = 0.620), pure muscle (*p* = 0.508), and intramuscular fat (*p* = 0.722) between the endoleak group and non-endoleak group, which reflects the endoleaks had no relationship to the imaging features of abdominal body composition. Therefore, the presence of endoleaks had no impact on the conclusions from the shape and gray feature analysis.

Considering the differences in BC between different genders and the limited number of female patients in this study, we further analyzed the shape and gray features of the enrolled male patients. Statistics showed that Group(−) still got a lower volume ratio of intramuscular fat **/** pure muscle (0.06 vs 0.08; *p* = 0.020) and a higher average CT value of total muscle tissue (31.43 HU vs 26.89 HU; *p* = 0.029). Analysis results of other imaging features also showed accordance with the data of total patients, which proved the credibility of our findings.

Compared with existing work, our research has the following characteristics. (a) Feature analysis based on consecutive multilayer images avoids the bias caused by the single-layer slice. (b) The detailed distinction of various BC facilitates a comprehensive understanding of the role of different tissues in aneurysm progression. (c) Analysis of multiple features and their mutual verification contribute to the comparison of abdominal BC from different aspects. (d) Few studies evaluated the relationship between gray features of BC and aneurysm growth. Our research proved that gray features are also associated with AAA progression and can make an early prediction.

There were several limitations to our work. (a) Only the first-order gray features are evaluated in this paper, and the second-order features such as texture are not further analyzed. (b) The flow of contrast medium is a dynamic process. Although we guarantee the same trigger conditions, it may still affect the evaluation of gray features. (c) Limited by the maturity of current segmentation technology, it is difficult to obtain shape features accurately and quickly. (d) For muscle tissues, the sample size in this study had adequate statistical power with an average beyond 75% and a maximum of 99%. However, for fat features, the maximum was only 48%. Thus, larger sample research is needed to further verify the finding of adipose tissue.

## Conclusions

In conclusion, muscle imaging features are associated with postoperative aneurysm progression. Patients with stable or shrunken aneurysms have a higher proportion of pure muscle with greater CT attenuation. Adipose tissue is not specifically related to AAA growth, although larger-scale studies are required to further confirm this finding. These results provide strong proof that increasing muscle–fat ratio or improving muscle mass and muscle quality, rather than simple weight reduction, can be more beneficial to patients after EVAR.

Furthermore, muscle imaging features can make an early prediction for aneurysm growth and obtain a higher accuracy of classification compared with traditional characteristics, such as endoleak. Therefore, imaging features (shape features and gray features) analysis of abdominal BC has the potential to become a new strategy for the prediction of postoperative AAA progression, which can play an important role in the surveillance of patients after surgery.

## Supplementary Information

**Additional file 1.** Supplementary document describes the details of conventional imaging evaluation procedures, CT acquisition protocols, and definition of kurtosis and skewness. Furthermore, original volume data and unadjusted average area and circumference of abdominal body composition are also provided.

## Data Availability

The datasets used and/or analyzed during the current study are available from the corresponding author on reasonable request.

## Abbreviations

AAA: Abdominal aortic aneurysm
BC: Body composition
EVAR: Endovascular aneurysm repair
LRM: Logistic regression model
