# Supplementary material for: The association of body composition with abdominal aortic aneurysm growth after endovascular aneurysm repair
Source: Insights Imaging. 2022 Apr 25;13:76. doi: 10.1186/s13244-022-01187-7 (PMC9038972; doi:10.1186/s13244-022-01187-7)
Supplement: Supplementary file 1 — Additional file 1. Supplementary document describes the details of conventional imaging evaluation procedures, CT acquisition protocols, and definition of kurtosis and skewness. Furthermore, original volume data and unadjusted average area and circumference of abdominal body composition are also provided. [file 13244_2022_1187_MOESM1_ESM.docx]

# ELECTRONIC SUPPLEMENTARY MATERIAL

## Conventional imaging evaluation procedures

The volume of the abdominal aortic aneurysm was evaluated by the three-dimensional post-processing software (Advanced Vessel Analysis, Philips Healthcare, Netherlands) on a dedicated workstation (IntelliSpace Portal Version 9.0.4, Philips Healthcare, Netherlands). The measurement range of the aneurysm volume was defined from the level of the lower renal artery to the level of bifurcation of the common iliac artery. The contour of the aneurysm sac was manually outlined by the brush tool, including the aorta, and calcification or intraluminal thrombus existed at the edge of the aneurysm body in the given measurement region. After segmenting, the software would automatically reconstruct the aneurysm and calculate the volume data.

## CT acquisition protocols

All follow-up examination images were contrast-enhanced CT scans of the abdominal and pelvis, which were performed using a first-generation dual-source CT (SOMATOM Definition, Siemens Healthcare, Germany). The institutional protocol was biphasic, a head-first supine position, and a scanning range from the diaphragm level to the pubic symphysis level. The contrast medium iopromide (370 mgI/ml, Shanghai Bolaike Xinyi Pharmaceutical Co., Ltd.) was injected using a high-pressure syringe through the median right elbow at a rate of 4.0 ml/s. A bolus tracking technique was used with a triggering threshold of 100 HU; the trigger level was the abdominal aorta. Other protocol parameters were as follows: tube voltage, 120 kV; tube current, 200 mAs; rack rotation time, 330 ms; collimation, 2 32 0.6 mm; pitch, 0.8 mm; the size of the two-dimensional slice, 512 × 512 pixels; slice thickness, 3-7 mm.

## Kurtosis and Skewness

Kurtosis is the peakedness or flatness of a frequency distribution, especially with respect to the concentration of values near the mean value as compared with the normal distribution. If kurtosis = 3, data is a normal distribution. If kurtosis > 3, data is leptokurtic. Otherwise, data is platykurtic. Skewness is a measure of the uniformity of distribution. If skewness = 0, data obeys normal distribution. If skewness > 0, data is positive-skewness or right-skewness distribution, which means more data is smaller than the mean value. Otherwise, data is negative-skewness or left-skewness distribution, which means more data is greater than the mean value.

# Supplementary Tables

## Supplementary Table S1 Original volume data of abdominal body composition

| **Volume (cm^3^)** | **All** | **Group(+)** | **Group(-)** |
| --- | --- | --- | --- |
| Subcutaneous fat | 1164.48 ± 458.26 | 1204.52 ± 403.10 | 1146.62 ± 482.72 |
| Visceral fat | 1507.45 ± 648.74 | 1582.41 ± 779.25 | 1474.01 ± 585.00 |
| Pure muscle | 1200.03 (1000.60, 1391.41) | 1077.83 (952.91, 1403.29) | 1232.35 (1054.23, 1387.08) |
| Intramuscular fat | 78.97 (54.71, 114.79) | 100.41 (67.95, 132.07) | 71.44 (50.17, 109.45) |
| Total fat | 2671.92 ± 993.76 | 2786.91 ± 1084.05 | 2620.62 ± 955.12 |
| Total muscle | 1286.68 (1114.82, 1473.85) | 1175.87 (1036.58, 1541.19) | 1317.36 (1131.54, 1467.68) |
| Abdomen | 5806.13 ± 1381.81 | 5903.77 ± 1548.62 | 5762.56 ± 1311.15 |

Unless otherwise specified, data are mean ± standard deviation or median (interquartile range). Group(+) represents patients with an aneurysm expansion and Group(-) represents patients with a stable or shrunken aneurysm.

## Supplementary Table S2 Unadjusted average area and circumference of body composition

| **Features** | **All** | **Group(+)** | **Group(-)** |
| --- | --- | --- | --- |
| **Average area (cm^2^)** | | | |
| Subcutaneous fat | 128.74 ± 44.98 | 133.82 ± 39.59 | 126.48 ± 47.30 |
| Visceral fat | 167.86 ± 69.12 | 174.87 ± 77.19 | 164.74 ± 65.60 |
| Pure muscle | 135.71 ± 22.12 | 132.65 ± 23.39 | 137.08 ± 21.58 |
| Intramuscular fat | 10.05 ± 5.18 | 11.98 ± 5.80 | 9.19 ± 4.67 |
| Total fat | 296.61 ± 102.07 | 308.69 ± 104.58 | 291.21 ± 101.28 |
| Total muscle | 145.76 ± 22.88 | 144.63 ± 24.73 | 146.27 ± 22.18 |
| Abdomen | 645.710 ± 122.86 | 659.77 ± 133.35 | 639.42 ± 118.42 |
| **Circumference (cm)** | | | |
| Abdomen | 97.5 ± 9.35 | 98.44 ± 9.78 | 97.09 ± 9.20 |

Unless otherwise specified, data are mean ± standard deviation or median (interquartile range). Group(+) represents patients with an aneurysm expansion and Group(-) represents patients with a stable or shrunken aneurysm.
